# A Seroprevalence Study of Toxoplasmosis in Female Students in Zahedan, South East of Iran

**Published:** 2019-05

**Authors:** Mohammad JAFARI-MODREK, Raheleh HASANZADEH, Hakim AZIZI, Kareem HATAM-NAHAVANDI

**Affiliations:** 1. Infectious Diseases and Tropical Medicine Research Center, Zahedan University of Medical Sciences, Zahedan, Iran; 2. Department of Parasitology and Mycology, School of Medicine, Zahedan University of Medical Sciences, Zahedan, Iran; 3. Department of Parasitology and Mycology, School of Medicine, Zabol University of Medical Sciences, Zabol, Iran; 4. Department of Microbiology and Immunology, School of Medicine, Iranshahr University of Medical Sciences, Iranshahr, Iran

## Dear Editor-in-Chief

*Toxoplasma gondii*, the causative agent of toxoplasmosis, is one of the more common parasitic zoonoses world-wide. Humans become primarily infected by oral ingestion of viable tissue cysts contained in undercooked meat, or by ingesting water or food contaminated with oocysts from infected feline faeces (1). Although, the infection with *T. gondii* is usually asymptomatic in immunocompetent humans, it can be fatal in the immunocompromised patients (1). Primary infection in a pregnant woman can also cause severe and disabling disease in the developing fetus (1).

The traditional diagnosis of toxoplasmosis routinely depends on bioassays and serological tests, such as ELISA, which rely on the detection of anti-*Toxoplasma* antibodies including IgG (indicating remote infection) or IgM (indicating acute infection) (1).

In present study, to achieve information regarding the point seroprevalence of *T. gondii* in Zahedan, Iran, a cross-sectional study was conducted in 2014 in the Girls’ Dormitory of Zahedan University of Medical Sciences (ZAUMS), Zahedan, Iran.

A total of 80 voluntary female students aged 18 to 24 yr were included in the study; sociodemographic characteristics of the individuals were recorded on the request forms. Blood samples were collected in collection tubes, and the tubes were then transferred to the serology laboratory at the department of parasitology and mycology of ZAUMS and kept at −20°C, until use. Serum samples obtained from whole blood were tested for anti-*Toxoplasma* IgG and IgM, using commercial ELISA kits (Pishtaz Teb Diagnostics, Iran) as detailed by manufacturer’s protocol. According to the manufacture’s announcement, the sensitivity and specificity of the kits were 100 and 99 percents, respectively. Sera were positive that titer of it was 1:200 and/or higher for IgG and IgM antibodies. SPSS version 20 (IBM SPSS Inc., Chicago, IL, USA) was used for statistical analyses and the significance level was set at 0.05.

Informed consent was taken from the participants before the study.

In this study, from the total of 80 blood samples, anti-*Toxoplasma* antibodies were detected in sera of 8 students, corresponding to an overall seroprevalence of 10% in this population, all seropositive for IgG. None of the samples were positive for IgM anti-toxoplasma antibodies. Among IgG antibody positive persons, 7 (87.5%) were residents of urban areas and 1 (12.5%) was rural. One (12.5%) of 8 IgG-antibody positive persons had contact with cat (Table 1). None of IgG-antibody positive persons had a history of eating raw meat (Table 1). No correlation was found between *Toxoplasma* seropositivity and contact with cat or history of eating raw meat (*P*<0.05). Moreover, no association was found between the resident in urban or rural area and toxoplasma seropositivity (*P*<0.05 on Chi-Square test) (Table 1).

**Table 1:** Seroprevalence of *T. gondii* infection, by selected sociodemographic characteristics, in female students in the girls’ dormitory of ZAUMS, 2014

***Characteristic***	***No. of subjects (%)***	***IgG positive (%)***	***P^a^***
Contact with cat			0.91
yes	11/80 (13.8)	1/8 (11.1)	
no	69/80 (86.2)	7/8 (87.5)	
History of eating raw meat			0.63
yes	2/80 (2.5)	0/8 (00.0)	
no	78/80 (97.5)	8/8 (100)	
Resident in urban or rural area			0.57
urban	74/80 (92.5)	7/8 (87.7)	
rural	6/80 (7.5)	1/8 (12.5)	

a Determined by Chi-Square test

Toxoplasmosis sero-surveys have been held in many countries (2). It is estimated that approximately 30–50% of the world population has anti-*Toxoplasma* antibodies (1, 2). Based on the results of a recent meta-analysis study, seroprevalence rate of toxoplasmosis in the Iranian general population was reported to be 39.3% (1). Results reported in our study correspond with those from study carried out in Bojnurd City (North Khorasan province) (3). The authors found 20.4% seropositive samples for IgG anti-*Toxoplasma* antibodies and no seropositivity for IgM anti-*Toxoplasma* antibodies in student girls. In Isfahan Province (the central Iran) and Robat-Karim District (Tehran Province), seropositivity for IgG anti-*toxoplasma* antibodies in high school girls aged 15–19 yr was 17.5% and 17.7%, respectively (2). In Fasa District (Fars Province), seropositivity for IgG anti-*toxoplasma* antibodies among high school girls aged between 14 to 19 years was 10.0% (2). In a study (4) in Jordan University of Science and Technology, the point seroprevalences of IgG and IgM antibodies among girl students aged between 18 to 23 yr was determined to be 66·5% and 0·5%, respectively. The results from each of these studies are not strictly comparable, and differences may be attributed to the geographical locations of the populations studied.

The present study presents reliable information about the distribution of *T. gondii* in southwest of Iran. However, further studies with more clinical samples may increase knowledge about the distribution of this parasite in Sistan and Baluchestan. The results of this study might help public healthcare systems in the management of toxoplasmosis.
